# Breath acidification in adolescent runners exposed to atmospheric pollution: A prospective, repeated measures observational study

**DOI:** 10.1186/1476-069X-7-10

**Published:** 2008-03-07

**Authors:** Jill M Ferdinands, Carol A Gotway Crawford, Roby Greenwald, David Van Sickle, Eric Hunter, W Gerald Teague

**Affiliations:** 1 Air Pollution and Respiratory Health Branch, Centers for Disease Control and Prevention, 1600 Clifton Road NE, MS A-32, Atlanta GA 30333, USA; 2 Office of Career and Workforce Development, Centers for Disease Control and Prevention, 1600 Clifton Road NE, MS E-94, Atlanta GA 30333, USA; 3 Emory Pediatrics Asthma Research Center, Department of Pediatrics, Emory University, 2015 Uppergate Drive, Atlanta GA 30322, USA; 4 Department of Population Health Sciences, University of Wisconsin School of Medicine and Public Health, 610 Walnut St, 707 WARF, Madison, WI 53726, USA

## Abstract

**Background:**

Vigorous outdoors exercise during an episode of air pollution might cause airway inflammation. The purpose of this study was to examine the effects of vigorous outdoor exercise during peak smog season on breath pH, a biomarker of airway inflammation, in adolescent athletes.

**Methods:**

We measured breath pH both pre- and post-exercise on ten days during peak smog season in 16 high school athletes engaged in daily long-distance running in a downwind suburb of Atlanta. The association of post-exercise breath pH with ambient ozone and particulate matter concentrations was tested with linear regression.

**Results:**

We collected 144 pre-exercise and 146 post-exercise breath samples from 16 runners (mean age 14.9 years, 56% male). Median pre-exercise breath pH was 7.58 (interquartile range: 6.90 to 7.86) and did not change significantly after exercise. We observed no significant association between ambient ozone or particulate matter and post-exercise breath pH. However both pre- and post-exercise breath pH were strikingly low in these athletes when compared to a control sample of 14 relatively sedentary healthy adults and to published values of breath pH in healthy subjects.

**Conclusion:**

Although we did not observe an acute effect of air pollution exposure during exercise on breath pH, breath pH was surprisingly low in this sample of otherwise healthy long-distance runners. We speculate that repetitive vigorous exercise may induce airway acidification.

## Background

Ground-level ozone and particulate matter (PM) are the primary ambient air pollutants of smog. Exposure to ozone causes airway irritation, wheezing, coughing, pain upon inspiration, and breathing difficulties, and repeated exposure may impair lung growth and cause permanent lung damage [1]. Exposure to PM has been repeatedly associated with increased mortality, although how PM causes death is not well understood [2]. For both ozone and PM, health risks rise as exposure rises.

Student athletes often exercise vigorously in smoggy outdoor environments, and athletic practices frequently occur in the mid- to late-afternoon when diurnal ozone levels are highest. Vigorous exercise increases minute ventilation and inspiratory flow rate, intensifying exposure of the distal lung to airborne pollutants. Although several studies have documented short- and long-term effects of ozone related to exposure during exercise [3-15], few have examined children or young adults, and little is known about effects of repeated exposures to ambient ozone or PM among student athletes.

Breath condensate contains a number of constituents derived from the respiratory surfaces that hold promise as indices of inflammation. The pH of exhaled breath condensate (EBC) has been proposed as a biomarker of inflammation reflecting the acid-base balance of the airways, which is regulated primarily by the proton-buffering effects of ammonium derived from the oral cavity and airways [16]. Several studies suggest that pH of exhaled breath condensate is low in various inflammatory lung diseases including poorly controlled asthma, COPD, cystic fibrosis, and acute lung injury [17-27].

Studies reporting negative correlations between EBC pH and pro-inflammatory cytokines in the airways [17,18] further suggest that EBC pH has promise as a biomarker of the early airway inflammation preceding frank symptoms or lung function impairment. Because it is relatively easy to collect and measure, breath pH has potential as an effective way to assess inflammatory effects of air pollution in high risk populations. Two studies have explored EBC constituents as biomarkers of respiratory morbidity from ozone exposure, including one that examined breath pH, finding no significant change in breath pH after ozone exposure [5,19].

The aim of the present study was to test the hypothesis that vigorous outdoor activity in student athletes exposed to summer air pollution might induce lung inflammation and thereby reduce breath pH. To accomplish this, we collected EBC pre- and post-exercise on ten days during peak smog season in 16 high school athletes engaged in daily long-distance running in a down-wind suburb of Atlanta.

## Materials and Methods

### Study design

This was a prospective observational study with a repeated measures design in which atmospheric concentrations of ozone and PM_2.5 _were the independent variables and pH of exhaled breath condensate (referred to as breath pH) was the primary outcome variable.

### Subjects

Sixteen members of a high school cross country team participated in the study. Participants had no history of respiratory infection in the four weeks prior to the study. Informed consent from parents of the participants and informed assent from the participants were obtained according to a protocol approved by the Institutional Review Boards of the Centers for Disease Control and Prevention and Emory University.

### Study procedures

Runners completed a health questionnaire at baseline and trained outdoors between 4 and 5 pm for ten days during a 15-day study period. Before and after training, study coordinators asked runners about respiratory symptoms, performed spirometry (EasyOne spirometer, ndd, Andover, MA), measured on-line exhaled nitric oxide at a flow rate of 50 mL/second (DENOX 88, EcoMedics, MI), and collected 5-minute breath condensate samples (Rtube^®^, Respiratory Research, Charlottesville, VA) from each participant. Breath condensate sample were collected during tidal breathing without use of noseclips. Spirometric results were expressed as the percent-predicted forced expiratory volume in 1 second (FEV_1_) according to population reference standards from NHANES III [20] and submitted to a clinician-investigator (WGT) daily for interpretation and safety monitoring.

### Breath condensate analysis

Breath condensate samples were placed on dry ice immediately post-collection, transported to the laboratory daily, and stored at -70°C until analysis. To measure breath pH, 100 μL of EBC was thawed to room temperature and, to remove carbonate, de-gassed with argon for 10 minutes (Orion Instruments, Baton Rouge, LA) until pH was constant. De-aerated breath pH values in healthy subjects are believed to range from 7.4 to 8.8 [21]. However, because there remains uncertainty about the range of normal breath pH, we conservatively defined as low any breath pH value less than 7.0.

### Exposure assessment

The study took place from August 16 to 31, 2004, during the peak "smog season" known *a priori *as a time of expected poor air quality for this region. Maximum 1- and 8-hour ozone concentrations and hourly concentrations of particulate matter ≤2.5 microns in diameter (PM_2.5_) were obtained from the Georgia Ambient Air Monitoring System. The nearest monitoring station was <1 mile from the study site for ozone and approximately 12 miles for PM_2.5_.

### Variable definition

We defined "number of symptoms in past 24 hours" as the number of positive responses to questions asking if the subject experienced wheeze, cough, watery eyes, runny nose, itchy or scratchy throat, or sneezing within 24 hours prior to the start of practice. Each day, athletes rated their perceived exertion during running on a scale from 1 (least vigorous) to 10 (most vigorous).

### Statistical analysis

Nonparametric rank-sum tests were used to examine differences in breath pH between groups. Mean breath pH values were adjusted for within-subject correlation, age, sex, race, and body mass index (BMI). For ease of interpretation, results are presented as raw breath pH values; however, statistical tests were performed using a nonparametric transform of the breath pH variable (see details in Additional file). A general linear mixed model was used to examine the association between ambient air pollutants and post-exercise breath pH, controlling for pre-exercise breath pH. Same-day 1-hr maximum ozone (ppb) and PM_2.5 _at 5 pm (μg/m^3^) were log-transformed for statistical analysis. Ozone and PM_2.5_concentrations were highly correlated, with raw and transformed Pearson correlation coefficients of 0.9 and 0.8, respectively. To avoid the difficulty of fitting a regression model with highly correlated independent variables, we ran separate models for ozone and PM_2.5_. Analyses were conducted in SAS version 9.1 (SAS Corporation, Cary NC) and SPSS for Windows (Version 14).

### Control group comparison

During preliminary data analyses, we observed frequent low breath pH values among the student athletes comprising the primary study sample. To investigate this unexpected result, we undertook a *post-hoc *comparison of resting breath pH values among the student athletes to breath pH values observed among a convenience sample of 14 healthy, non-smoking sedentary adults recruited from the faculty and staff of the CDC and Emory University. These adult subjects provided resting breath condensate samples both indoors and outdoors at 4:00 pm on two days with relatively low concentrations of air pollutants. Each control subject provided a total of two breath samples, one taken indoors and one outdoors. To facilitate comparison of breath pH values between control subjects and the athletes, we used only breath samples taken outdoors from the controls (for a total of 14 control samples) and compared them to the subject-specific median pre-exercise breath pH values among the athletes using the nonparametric Mann-Whitney *U *test.

## Results

Mean age of participants (n = 16) was 14.9 years, 56% were male, and 69% were white (Table 1). Thirteen of 16 (81%) participants were distance runners and three were sprinters. Two of 16 (13%) reported having asthma, one of whom participated in only the first three days of the study. None were smokers. From 16 participants, we collected 144 pre-exercise and 146 post-exercise breath samples.

**Table 1:** Selected characteristics of study participants (n = 16 subjects).

**Variable**	**Value**
Age, yr	
Mean (SD)	14.9 (0.9)
Range	14–17
Body mass index, kg/m^2^	
Mean (SD)	19.8 (1.7)
Range	17.5 – 23.5
Sex, N(%)	
Male	9 (56)
Female	7 (44)
Race, N(%)	
White	11 (69)
Nonwhite	5 (31)
Self-reported asthma diagnosis, N(%)	
Yes	2 (13)
No	14 (88)
Self-reported allergy diagnosis, N(%)	
Yes	4 (25)
No	12 (75)
Wheeze or cough in past month, N(%)	
Yes	4 (25)
No	12 (75)
Home environmental tobacco smoke exposure (ETS), N(%)	
Yes	2 (12)
No	14 (88)
Running style, N(%)	
Sprinter	3 (19)
Distance	13 (81)

Mean (± SD) ambient 1-hour maximum ozone concentration was 71 (18) ppb. Median (interquartile range, IQR) was 61 (54–67) ppb. Mean (± SD) PM_2.5 _measured at 5 pm was 27.2 (11.9) μg/m^3^. Median (IQR) was 23.2 (21.7 – 34.7) μg/m^3^. Four of 10 study days were air quality alert days, three of these triggered by high ozone levels and one by high PM levels. On three study days ozone concentrations exceeded health-based standards.

### Pre-exercise breath pH

**Figure 1:**
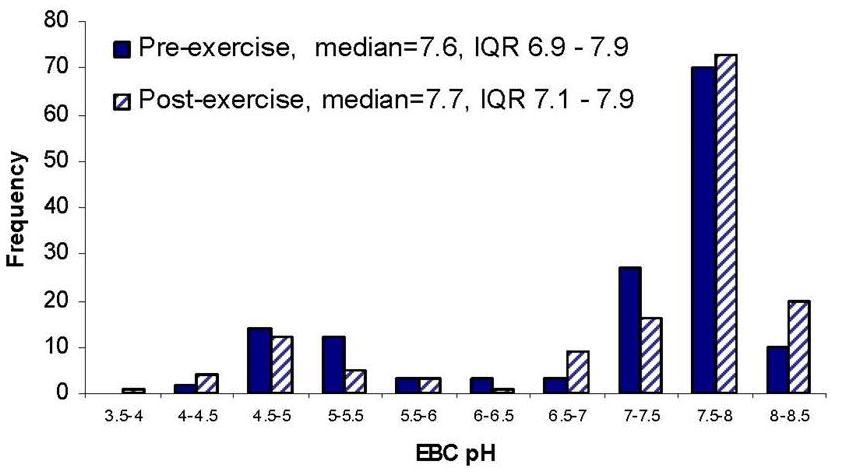
**Distribution of pre- and post-exercise breath pH values in runners**. In this group of adolescent runners, there was no significant difference between pre- and post-exercise breath pH (p = 0.63 for nonparametric test of difference in pre- and post-exercise breath pH distributions).

**Figure 2:**
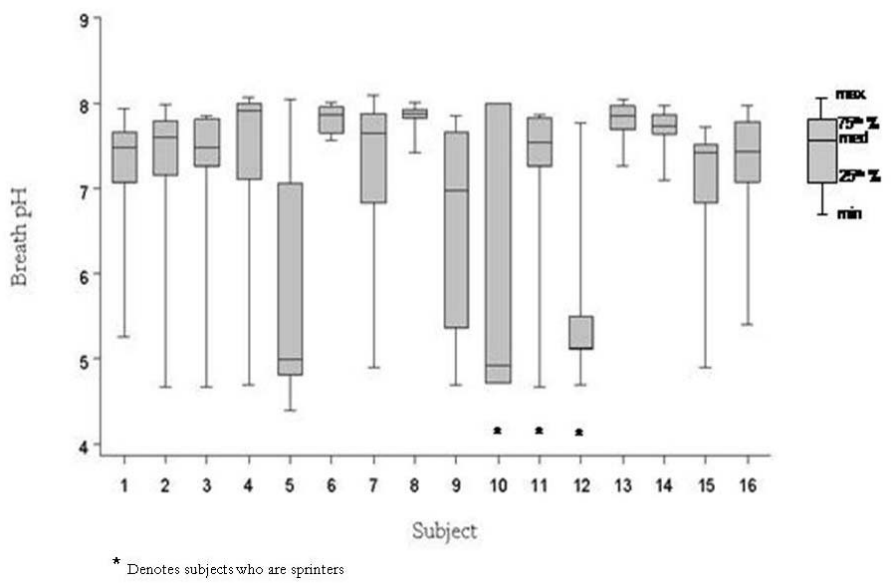
Pre-exercise breath pH by subject (n = 16 subjects, 144 samples), with sprinters denoted by asterisks.

Median pre-exercise breath pH was 7.58; range was 4.39 – 8.09; IQR was 6.90 – 7.86. Crude pre-exercise pH was lower among participants with home tobacco smoke exposure, those 16 to 17 years old, and sprinters (Table 2). Pre-exercise breath pH was bimodally distributed with a prominent peak around 7.5 and a smaller, unexpected peak around 5.0 (Figure 1). Individual median pre-exercise breath pH ranged from 4.9 to 7.9, with four subjects having median pre-exercise breath pH less than 7.0. Pre-exercise breath pH values varied considerably within subject over the ten study days (Figure 2), with day to day within-subject coefficient of variation ranging from 2 to 25%. Overall within-subjection coefficient of variation was 16%. Of 16 participants, 12 (75%) had at least one pre-exercise breath pH ≤ 6.0, and 10 (63%) had at least one pre-exercise breath pH ≤ 5.0.

**Table 2:** Median pre-exercise breath pH values for selected sample characteristics (n = 144 observations from 16 subjects).

**Variable**	**Obs (n)**	**Median (IQR) (*p value*)†**
Age, yr		
14	59	7.56 (7.05–7.87)
15	59	7.70 (7.34–7.86)
16–17	26	7.22 (5.12–7.58)
		(*0.01*)
BMI, kg/m^2^		
<20	90	7.68 (7.06–7.87)
≥20	54	7.41 (6.05–7.77)
		(*0.17*)
Sex		
Male	86	7.58 (7.05–7.85)
Female	58	7.56 (6.37–7.86)
		(*0.71*)
Race		
White	103	7.60 (7.10–7.87)
Nonwhite	41	7.44 (5.49–7.78)
		(*0.13*)
Asthma		
Yes	13	7.41 (5.00–7.51)
No	131	7.60 (7.05–7.86)
		(*0.10*)
Allergy		
Yes	40	7.51 (7.27–7.82)
No	104	7.60 (6.01–7.86)
		(*0.20*)
Wheeze or cough in past month		
Yes	33	7.51 (7.25–7.78)
No	111	7.59 (6.37–7.86)
		(*0.68*)
Number of symptoms in past 24 hours*		
0	116	7.55 (6.83–7.86)
1	22	7.68 (5.96–7.87)
2 or more	6	7.57 (7.27–7.66)
		(*0.58*)
Home ETS exposure		
Yes	20	7.06 (4.99–7.74)
No	124	7.60 (7.10–7.86)
		(*0.009*)
Running style		
Sprinter	21	6.05 (5.12–7.59)
Distance	123	7.64 (7.10–7.86)
		(*0.0004*)

* Symptoms included wheeze, cough, scratchy or itchy throat, runny nose, sneezing, and watery eyes.

IQR = interquartile range.

† Nonparametric Wilcoxon rank-sum tests used to examine differences in raw breath pH between groups.

Lower-than-expected values of pre-exercise breath pH (<7.0) were observed on all study days. At least one subject had pre-exercise breath pH ≤ 5.5 each day, although the particular subject varied day to day. On eight of ten study days, at least one participant had pre-exercise breath pH ≤ 5.0. Breath pH was not associated with date of assay or elapsed time between sample collection and assay.

### Post-exercise breath pH

Median post-exercise breath pH was 7.68; range was 3.78 – 8.17; IQR was 7.05 – 7.92. Breath pH did not change significantly after exercise (p = 0.31 by paired *t*-test or *t *test of difference).

We observed no statistically significant association between post-exercise breath pH and same-day 1-hr maximum ozone concentration, controlling for race, home tobacco smoke exposure, perceived exertion during running, and pre-exercise breath pH (see Additional file 1, Table 1). Similarly, we observed no statistically significant association between post-exercise breath pH and same-day PM_2.5 _at 5 pm, controlling for the same factors (see Additional file 1, Table 2). No significant associations were observed when ozone and PM_2.5 _concentrations were lagged by 1 or 2 days. Results were not qualitatively different in models that used transformed breath pH or transformed hydrogen ion concentration variables instead of raw breath pH variables. Adjusted post-exercise breath pH was lower among nonwhite subjects compared to white subjects (p < 0.01 in both ozone and particulate models) and among subjects reporting no home tobacco smoke exposures (p < 0.001 in ozone model, p < 0.01 in PM_2.5 _model). As expected, post-exercise breath pH was positively associated with pre-exercise breath pH (p < 0.05 in all models). Resting lung function (measured as percent-predicted FEV_1_) was not associated with resting breath pH, and post-exercise lung function was not associated with ozone or PM_2.5_concentrations (results not shown). Exhaled nitric oxide (ppb) was not significantly correlated with resting breath pH (results not shown; detailed analysis of exhaled nitric oxide to be reported separately).

**Figure 3:**
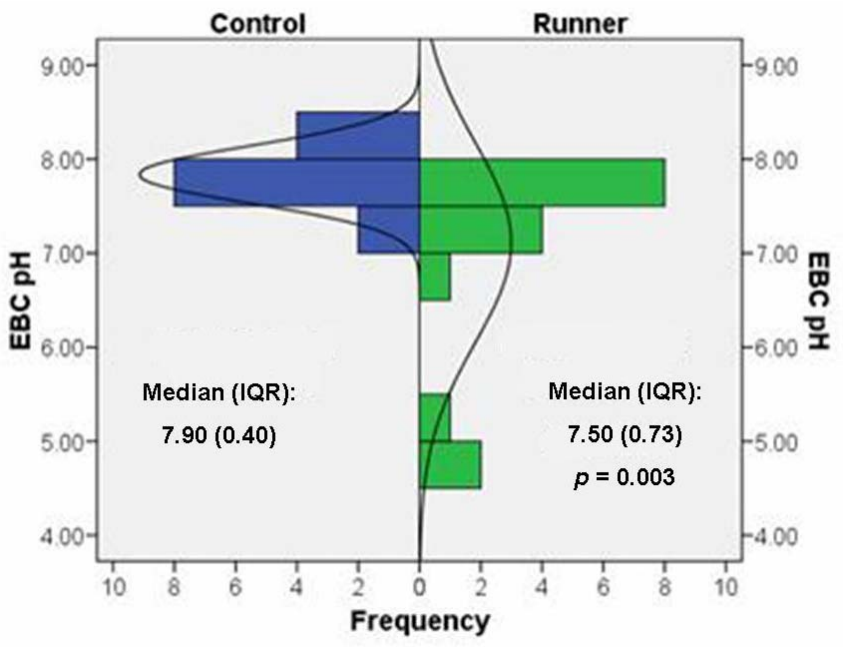
**Outdoor resting breath pH was lower in runners (n = 16; green) compared to controls (n = 14; blue)**. Observed outdoor resting breath pH in this group of adolescent runners was lower than that seen in a control group of non-smoking, healthy adults, none of whom were involved in regular long-distance outdoor running (p = 0.003 by Mann-Whitney *U *test).

Among the *post-hoc *control group of fourteen healthy adults not engaged in daily long-distance running, no unusually low breath pH values were observed (Figure 3). The median breath pH of 7.50 observed among the student athletes was significantly lower than median breath pH of 7.90 seen in the controls (p = 0.003). Variation in breath pH was substantially greater among the student athletes (interquartile range of 0.73) than among sedentary adult controls (interquartile range of 0.40). (Note that the median breath pH of 7.50 among the student athletes reported here is slightly different from the median of 7.58 reported above because the former is calculated from subject-specific medians for each athlete rather than the 144 crude breath pH values). In the *post-hoc *adult controls, we observed no clear pattern of differences between indoor and outdoor breath pH levels and therefore report only the outdoor values here to facilitate comparison to the runners.

## Discussion

In this study, we sought to examine the association of exhaled breath pH with ambient ozone and particulate matter concentrations among 16 adolescent athletes exposed to these air pollutants during vigorous outdoor exercise. Although there was considerable variation in air quality over the 10 study days, we did not observe a relationship between air pollution levels and post-exercise breath pH. Overall, post-exercise breath pH was indistinguishable from pre-exercise breath pH, suggesting no acute effect of air pollution exposure during vigorous outdoor exercise on breath pH compared to resting values. This finding is similar to that of Corradi et al [5], who found no change in EBC pH after short-term exposure to ozone during light exercise and is the only study apart from ours that examined the association between air pollution exposure during exercise and EBC pH.

Although we did not observe an acute effect of outdoor exercise, we found that both resting and post-exercise breath pH values were intermittently lower than expected in a majority of subjects. Although airway pH in healthy individuals is believed to be slightly alkaline with pH of 7–8 [21,22], we observed in a majority of subjects intermittent breath pH values comparable to levels seen in severe inflammatory conditions such as acute asthma and sickle cell anemia. Day-to-day variability in breath pH was common and similar in magnitude to that seen in children with persistent asthma. We observed much greater within-subject variation in breath pH than previously reported [17,22-25]. The intermittently low breath pH values we observed were not correlated with subject, study day, or assay date, and resampling of banked samples suggested that they could not be attributed to random laboratory error.

Potential explanations for the low breath pH values that we observed include the possibility that salivary contamination (for instance, from gastroesophageal reflux) or other artifact of sample collection (such as recent eating or drinking) artificially lowered breath pH. From a mechanistic standpoint, it may be possible that routine vigorous exercise and its concurrent effect on systemic lactate production influenced airway pH. Because EBC samples were collected outdoors, it is plausible that inhaled atmospheric sulfates reduced EBC pH. Alternatively, it is possible that these otherwise healthy athletes experienced significant intermittent airway acidification events that were accurately reflected by the observed low breath pH values. We address each of these possibilities in turn.

Our use of a one-way valve system with saliva trap during EBC collection makes it unlikely that salivary contamination was responsible for the low pH values [21], and a previous study of the potential for salivary contamination using the same collection system showed no such contamination [22]. Although we cannot rule out that the athletes had eaten in the hour prior to collection of the resting breath samples, it is unlikely because samples were collected immediately after the students were dismissed from final classes of the day. Furthermore, the subjects did not eat during running, so we are confident that the low post-exercise breath pH values are not attributable to having eaten. Exposure to ingested liquids was restricted to water and non-carbonated sports drinks. Although it is plausible that sports drink consumption could contribute to acidic breath pH, three observations make us doubt that this is the case: (1) the principal investigator was present during each breath sampling period and observed only a small number of students consuming sports beverages, far fewer than the 75% with acidic breath pH values; (2) presumably more sports drinks would have been consumed after vigorous exercise, but acidic breath pH values were observed with similar frequency before and after exercise; (3) in a recent re-analysis of the breath pH samples, we identified citric acid – a common ingredient in sports drinks – in only six breath samples, suggesting that few athletes consumed sports drinks. Moreover, pH was normal in the breath samples containing citric acid.

Another possible explanation for the observed low pH values is the potential effect of gastroesophageal reflux on breath pH. It is plausible that this acid reflux is stimulated by vigorous exercise and may contribute to intermittent breath pH acidification events via salivary contamination. If this were the case, however, we would expect more frequent breath acidification events following vigorous exercise. On the contrary, we saw similar frequencies of acidification events before and after exercise. It is conceivable that reflux may have been stimulated by physical activity prior to practice, for instance if athletes were rushing from class to the practice field. We have no evidence to confirm or refute this possibility other than anecdotal recollection suggesting that this level of pre-practice activity was unlikely to have occurred often enough to account for the frequency with which acidic pre-practice breath pH values were observed. Also, as noted above, salivary contamination is unlikely due to the design of the Rtube^® ^breath collection system. Nonetheless, acid reflux as a contributor to the observed low breath pH values cannot be ruled out definitively.

Because (EBC) samples were collected outdoors, we explored the possibility that the low breath pH values we observed were artifacts of outdoor sample collection (rather than accurate reflections of airway pH). Outdoor sample collection could conceivably lower condensate pH if inhaled ambient sulfate particles did not deposit in the respiratory tract but were rather collected in the breath condensate. To examine this possibility, we constructed a simple mathematical model to estimate the maximum amount of sulfate expected in the condensate samples based on the concentration of sulfate and other relevant atmospheric ions in the ambient air (see Additional file 1). We also estimated the predicted breath pH and compared it to measured breath pH. These comparisons were conducted on a subsample of five runners on two study days, one chosen to represent a day with high ambient sulfate levels and the other a day with low ambient sulfate levels. The results of this modeling (see Additional file 1, Table 3) suggest that inhaled ambient sulfate particles may potentially lower breath pH by a small amount, but this effect is minimal and unlikely to explain entirely the surprisingly low breath pH values we observed in the student athletes.

**Table 3:** Studies reporting breath pH among healthy subjects.

**Reference**	**Subjects**	**Central tendency**	**Range**	**CV**	**Comments**
***Children:***					
Carpagnano et al 2004. [23]	15 healthy children with mean age 7 yr	Mean (SE): 7.85 (0.02)	NR; estimated 7.6 – 8.2 from Figure 1A	CV = 0.04% from 6 adult controls	Samples de-aerated
Carraro et al 2005 [31]	10 healthy children with mean age 10 yr	Median (IQR): 7.85 (7.80 – 7.90)	NR; estimated 7.7 – 8.0 from Fig 2	NR	Samples de-aerated
MacGregor et al 2005 [32]	47 healthy control children of mean age 16 yr	Median: 5.90	5.00–7.30	NR	Samples not de-aerated
Nicolaou et al 2006 [30]	562 8-year old children from unselected population-based birth cohort	Median (IQR): 7.77 (7.59–7.87)	Range for normal subjects not reported. For all subjects (including 54 asthmatics and 562 normals): 4.40–8.29	NR	Bimodal distribution that "could not be normalized"; samples de-aerated
Rosias et al 2004 [33]	9 control children with mean age 9 yr	Median (SEM): 8.11 (0.07) de-aerated 6.64 (0.05) non-de-aerated	NR	NR	pH reported both before and after de-aeration
***Adults:***					
Borrill et al 2005 [34]	12 healthy adults with mean age 26 yr	Mean (95%CI): 7.61 (7.52 – 7.70)	NR	NR^a^	Samples de-aerated
Carpagnano et al 2005a [24]	15 healthy adults with mean age 35 yr	Mean (SD): 7.85 (0.14)	NR; estimated 7.6–8.2 from Fig IC	CV = 0.4% in 10 healthy adults	Samples de-aerated
Carpagnano et al 2005b [17]	7 healthy adults with mean age 42 yr	Mean (SEM): 7.9 (0.1)	NR; Estimated 7.8–8.2 from Fig 3C	CV = 0.4% in 10 healthy adults^b^	Samples de-aerated
Corradi et al 2002. [5]	22 healthy adults with mean age 30 yr, grouped by *NQO1* and *GSTM1* genotype	Mean^c^: Group 1 (*NQO1*wild type and *GSTM1*null): 7.91 Group 2 (all other genotypes): 8.01	Group 1 (*NQO1*wild type and *GSTM1 *null): 7.70 – 8.08 Group 2 (all other genotypes): 7.80–8.11	NR	Samples apparently de-aerated following procedures in Hunt 2001
Gessner et al 2003 [35]	12 healthy adults with mean age 57	Mean (SD): 7.46 (0.48)	NR	NR	Samples de-aerated
Hunt et al 2000 [25]	19 healthy subjects with mean age 20.5	Mean (SE): 7.65 (0.20)	NR; estimated 4.6 – 8.5 from Fig 1	CV = 3.3% from 6 normals and 3 asthmatics; CV in normals not reported	Samples de-aerated
Kostikas et al 2002 [18]	10 healthy adult controls with mean age 34 yr	Mean (95%CI): 7.57 (7.51–7.60)	NR; estimated 7.4 – 7.75 from Fig 1A	NR	Samples de-aerated
Niimi et al 2004 [36]	16 healthy adults with mean age 43 yr	Mean (SD): 8.26 (0.20)	NR; estimated 7.8 – 8.6 from Fig 1	NR	Samples de-aerated
Ojoo et al 2005 [37]	15 healthy adults with mean age 39 yr	Median (IQR): 6.08 (5.58–6.64)	NR; estimated 5.6 – 6.7 from Fig 2	NR	Samples not de-aerated
Paget-Brown et al 2006 [29]	404 healthy children and adults from 0 to >71 yr of age	Mean: 7.85 Median (IQR): 8.0 (7.8 – 8.1); In 11 to 20 yr olds (n = 163): Median (IQR): 8.0(7.8–8.1) Mean (SD): 7.8 (0.7)	4.5–8.4	NR	Samples de-aerated
Tate et al 2002 [38]	12 healthy adults with mean age 33 yr	Mean (SD): 6.15 (0.16)	NR; estimated 5.8 – 6.5 from Fig. 1	NR^d^	Samples not de-aerated
Vaughan et al 2003 [22]	76 healthy adults with mean age 21 yr	Mean (SD): 7.70 (0.49)	NR	Mean CV = 4.5%; by subject, CV = 0.9 – 20%	Samples de-aerated

CV = coefficient of variation

NR = not reported

^a ^reports "limits of agreement" using Bland-Altman methods to assess reproducibility

^b ^appears to be reporting the same CV estimate as in Carpagnano 2005a

^c ^breath pH measurement at baseline (before exposure to ozone)

^d ^reports "coefficient of acceptability" as 0.08 pH units

Why we observed low and highly variable breath pH values in this group of otherwise healthy athletes is unclear. One possibility is that the routine vigorous exercise reported by these athletes caused systemic lactic acid production that altered resting airway pH. The mechanism by which the respiratory and systemic acid-base buffering systems may interact to cause this effect is poorly understood and requires more study. Another potential explanation is that long-term exposure to outdoor air pollution – such as may be experienced by children who routinely exercise outside during polluted summers – may trigger intermittent endogenous airway acidification events indicative of pollution-related lung inflammation. This idea is broadly consistent with results of several studies that suggest that long-term exposure to urban air pollution may cause lung damage or lung growth impairment in children [4,26-28] and could partially explain why we did not see similar values among a comparison group of healthy but relatively sedentary adults. Another possibility is that low pH values representing endogenous intermittent airway acidification events are normal rather than pathologic phenomena. Supporting this speculation are several recent studies that measured breath pH among healthy controls (Table 3) and found surprisingly low breath pH in a subset of subjects [25,29], including one large population-based study that found breath pH values as low as 4.4 in healthy children [30]. The possibility that intermittent low breath pH values reflect normal biologic processes calls into question the utility of breath pH measurement – especially one-time breath pH measurement – as a reliable biomarker of lung inflammation or respiratory health effects.

This analysis has several limitations. The small number of subjects (n = 16) may have limited statistical power to detect relationships between ambient air pollutants and post-exercise breath pH. We had no data from personal monitoring of air pollutant exposure or on long-term exercise patterns or residential histories of the subjects and, thus, were unable to examine if ambient air pollution or low breath pH values were associated with cumulative exposure to polluted environments. That we did not see similar low breath pH values in a group of healthy adults with breath samples taken outdoors suggests that the low breath pH values observed among the student athletes may be attributable to characteristics of their lungs that we were unable to measure in this study. Because characteristics of our study subjects may have varied from those of student athletes in general, we caution against generalizing our results.

## Conclusion

In summary, we found no acute effect on breath pH from vigorous outdoor exercise on days with significant ozone and PM pollution in this group of 16 student athletes. We did, however, observe highly variable and surprisingly low breath pH values in a majority of these student athletes. These intermittent, low breath pH values are unexplained and may represent endogenous airway acidification. Additional studies with greater statistical power are necessary to rigorously examine the potential effect of outdoor exercise in polluted environments on the lungs of young athletes. However, before additional studies propose to use the pH of exhaled breath condensate as a biomarker of airway inflammation or respiratory morbidity, it will be necessary to determine if the low breath pH values we and others have observed among healthy individuals are indicative of respiratory pathology or normal biologic phenomena.

## Abbreviations

Akaike's Information Criterion (AIC), body mass index (BMI), Centers for Disease Control and Prevention (CDC), exhaled breath condensate (EBC), environmental tobacco smoke (ETS), forced expiratory volume in 1 second (FEV_1_), interquartile range (IQR), National Health and Nutrition Examination Survey (NHANES), not reported (NR), particulate matter (PM), particulate matter ≤2.5 microns in diameter (PM_2.5_), parts per billion (ppb), standard deviation (SD), standard error (SE), standard error of the mean (SEM)

## Competing interests

The author(s) declare that they have no competing interests.

## Authors' contributions

JF conceived of, designed, and coordinated the study, collected and analyzed data, and prepared the manuscript. CG performed statistical analysis and provided statistical expertise. RG participated in study design, data collection, data analysis, and manuscript preparation. DV participated in the design of the study, data collection, and data analysis. EH carried out laboratory analyses. WGT participated in study design, coordination, data collection, data analysis, and manuscript preparation. All authors read and approved the final manuscript.

## Supplementary Material

Additional file 1 Ferdinands et al. Breath acidification in adolescent runners: additional information. This file provides (a) detailed description and results of statistical methods used in the analysis; and (b) detailed description and results of modeling and analytic methods used to predict sulfate concentration and pH of exhaled breath condensate based on ambient air concentrations, as well as methods used compare those to observed sulfate concentration and pH of exhaled breath condensate. Click here for file
