# Supplementary material for: Breath acidification in adolescent runners exposed to atmospheric pollution: A prospective, repeated measures observational study
Source: Environ Health. 2008 Mar 7;7:10. doi: 10.1186/1476-069X-7-10 (PMC2292713; doi:10.1186/1476-069X-7-10)
Supplement: Additional file 1 — Ferdinands et al. Breath acidification in adolescent runners: additional information. This file provides (a) detailed description and results of statistical methods used in the analysis; and (b) detailed description and results of modeling and analytic methods used to predict sulfate concentration and pH of exhaled breath condensate based on ambient air concentrations, as well as methods used compare those to observed sulfate concentration and pH of exhaled breath condensate. [file 1476-069X-7-10-S1.doc]

**Additional File 1:** Ferdinands et al. Breath acidification in adolescent runners: additional information

**Statistical analysis.** Nonparametric Wilcoxon rank-sum tests were used to examine differences in breath pH between groups. A linear mixed model was used to examine the association between ambient air pollutants and post-exercise breath pH. Same-day 1-hr maximum ozone (ppb) and PM2.5 at 5pm (μg/m3) were the primary predictors of interest and were log-transformed for statistical analysis. Ozone and PM2.5 concentrations were highly correlated, with raw and transformed Pearson correlation coefficients of 0.9 and 0.8, respectively. To avoid the difficulty of fitting a regression model with highly correlated independent variables, we ran separate models for ozone and PM2.5. Model results are presented in Supplemental Tables 1 and 2.

The linear mixed model used to examine relationships between breath pH and air pollution levels accounted for correlation within subjects. Several correlation structures were examined; results differed little between structures. Because a first-order autoregressive structure yielded the lowest AIC (Akaike’s Information Criterion, a measure of goodness of fit), this structure was used. No random effects were assumed.

The asymmetric, bimodal distribution of observed breath pH was not substantially improved by typical mathematical transformations. We examined several methods for handling this lack of normality including using raw breath pH values, a natural log transform, raw breath pH values with a log link function, and a nonparametric transform that maps quantiles of the observed distribution to quantiles of a normal distribution, thus forcing a transformed variable that is normally distributed but lacking in substantive interpretation (referred to as the *normal score transform*). In simple bivariate models in which breath pH was regressed on each predictor individually, the sign of the regression coefficient (i.e., direction of the relationship) was the same across all methods. *P* values differed somewhat, with those estimated using the normal score transform not consistently larger or smaller than those estimated using the other methods. In only one instance was the *p* value estimated from the normal score transform greater than 0.05 while the *p* values estimated from the other methods was less than 0.05. Otherwise, the magnitude and significance of bivariate relationships did not qualitatively differ across methods, suggesting the models are robust to violation of normality. In multivariate models, the magnitude of regression coefficients was similar across methods and *p* values were generally lower using the normal score transform. To aid interpretation of model results, we present multiple models that are identical except for the outcome variable, including a model using raw breath pH as the outcome and an analogous model using transformed breath pH as the outcome.

To isolate the association between exposure to ambient air pollution during exercise and post-exercise breath pH, we controlled for pre-exercise breath pH in each model. We examined models in which we controlled for pre-exercise breath pH with a continuous variable or with a set of indicator variables, and results were essentially similar with either method. We also examined models in which the ozone and PM2.5 concentrations were lagged by one and two days, and results were qualitatively similar to the models in which no lag was assumed. Models using the appropriately transformed value of hydrogen ion concentration rather than pH as the outcome yielded qualitatively similar results (although direction of the associations was reversed, as would be expected given the relationship between pH and hydrogen ion concentration). Analyses were conducted in SAS version 9.1 (SAS Corporation, Cary NC).

**Breath sulfate analysis**. Because exhaled breath condensate (EBC) samples were collected outdoors, we explored the possibility that the low breath pH values we observed were artifacts of outdoor sample collection (rather than accurate reflections of airway pH). Outdoor sample collection could conceivably lower condensate pH if inhaled ambient sulfate particles did not deposit in the respiratory tract but were rather collected in the breath condensate.

To examine this possibility, we constructed a simple mathematical model to estimate the maximum amount of sulfate expected in the condensate samples based on the concentration of sulfate and other relevant atmospheric ions in the ambient air. To estimate the maximum possible effect, we assumed that none of the inhaled sulfate particles would deposit on respiratory surfaces but would instead be immediately exhaled and collected in the breath condensate with 100% efficiency. We furthermore assumed a constant minute ventilation of 3 liters/minute for ten minutes and a resultant EBC volume of 1 mL. Finally, we assumed there was no endogenous sulfate in the breath samples. We selected a high sulfate day and a low sulfate day from the air quality database, and then calculated the hypothetical molar concentration of sulfate assuming all the sulfate in 30 liters of air (3 liters/minute x ten minutes) was collected in 1 mL of water. The same procedure was repeated for other aerosol species in the air quality database, namely nitrate and ammonium. The actual concentration of sulfate, nitrate and ammonium in a subsample of five runners was subsequently measured using ion exchange chromatography. Since our assumptions of no particle deposition in the airways and no endogenous sulfate production are admittedly incorrect, the predicted and measured concentrations are not in agreement, although they are of the same order of magnitude. An estimation of breath pH was calculated using an iterative approach by estimating the proton concentration necessary to achieve ion balance with ammonium, sulfate and nitrate in our hypothetical 1 mL condensate sample based on the ambient atmospheric concentrations of ammonium, sulfate and nitrate reported in the air quality database.

The results of this modeling (see Additional Table 3) suggest that inhaled ambient sulfate particles may potentially lower breath pH by a small amount, but this effect is minimal and unlikely to explain entirely the surprisingly low breath pH values we observed in the student athletes.

**Additional Table 1. Estimated regression coefficients from mixed linear models† with post-exercise breath pH as outcome and 1-hour ozone concentration as ambient air pollutant predictor (n=143 observations from 16 subjects)**

| **Outcome variable** | **Model 1** | **Model 2** | **Model 3** | **Model 4** |
| --- | --- | --- | --- | --- |
|  | Raw post-exercise breath pH | Normal score transform of post-exercise breath pH | Raw post-exercise  breath pH | Raw post-exercise  breath pH |
| **Lag time** | Same day | Same day | 1 day lag | 2 day lag |
| **AIC criterion** | 409.1 | 371.5 | 411.4 | 412.7 |
| **Estimated regression coefficient, β (standard error):** | | | | |
| Race  Nonwhite  White | -1.06 (0.20)***  Ref | -0.64 (0.17)**  Ref | -1.05 (0.21)***  Ref | -1.05 (0.21)***  Ref |
| Home ETS exposure  Yes  No | 1.15 (0.27)***  Ref | 0.99 (0.23)***  Ref | 1.15 (0.27)**  Ref | 1.15 (0.27)***  Ref |
| Exertion (1 to 10 scale) | 0.04 (0.05) | 0.08 (0.04)* | 0.06 (0.05) | 0.06 (0.05) |
| Raw pre-exercise  breath pH | 0.18 (0.08)* | ---- | 0.19 (0.08)* | 0.19 (0.08)* |
| Normal score transform of pre-exercise breath pH | ---- | 0.18 (0.08)* | ---- | ---- |
| Same-day log max 1-hr ozone (log ppb) | 0.67 (0.37) | 0.50 (0.32) | ---- | ---- |
| 1-day lagged log max 1-hr ozone (log ppb) | ---- | ---- | 0.34 (0.28) | ---- |
| 2-day lagged log max 1-hr ozone (log ppb) | ---- | ---- | ---- | 0.04 (0.31) |

† All models use a first-order autoregressive correlation structure

* p<0.05

** p<0.01

*** p<0.001

**Additional Table 2. Estimated regression coefficients from mixed linear models† with post-exercise breath pH as outcome and PM2.5 concentration as ambient air pollutant predictor (n=143 from 16 subjects)**

| **Outcome variable** | **Model 1** | **Model 2** | **Model 3** | **Model 4** |
| --- | --- | --- | --- | --- |
|  | Raw post-exercise breath pH | Normal score transform of post-exercise breath pH | Raw post-exercise  breath pH | Raw post-exercise  breath pH |
| **Lag time** | Same day | Same day | 1 day lag | 2 day lag |
| **AIC criterion** | 413.7 | 375.0 | 412.5 | 413.2 |
| **Estimated regression coefficient, β (standard error):** | | | | |
| Race  Nonwhite  White | -1.05 (0.21)***  Ref | -0.63 (0.18)**  Ref | -1.05 (0.21)***  Ref | -1.05 (0.21)***  Ref |
| Home ETS exposure  Yes  No | 1.15 (0.27)***  Ref | 0.97(0.24)**  Ref | 1.15 (0.28)**  Ref | 1.15 (0.27)***  Ref |
| Exertion (1 to 10 scale) | 0.06 (0.05) | 0.10 (0.04)* | 0.07 (0.05) | 0.06 (0.05) |
| Raw pre-exercise  breath pH | 0.19 (0.08)* | ---- | 0.19 (0.08)* | 0.19 (0.08)* |
| Normal score transform of pre-exercise breath pH | ---- | 0.18 (0.08)* | ---- | ---- |
| Same-day log PM2.5 at 5pm (log μg/m3) | 0.05 (0.18) | -0.10 (0.16) | ---- | ---- |
| 1-day lagged log PM2.5 at 5pm (log μg/m3) | ---- | ---- | 0.21 (0.18) | ---- |
| 2-day lagged log PM2.5 at 5pm (log μg/m3) | ---- | ---- | ---- | 0.14 (0.18) |

† All models use a first-order autoregressive correlation structure

* p<0.05

** p<0.01

*** p<0.001

**Additional Table 3. Predicted and measured breath condensate sulfate concentrations and pH in a subsample of five runners**

|  | Atmospheric Ionic Species* (all μg/m3) | | | Breath Sulfate  Predicted/Measured**  μM | Breath pH  Predicted/Measured |
| --- | --- | --- | --- | --- | --- |
|  | SO­4­ 2- | NH­4­ + | NO­3­- |  |  |
| Study Day 4  (high sulfate day) | 14.98 | 4.86 | 0.86 | 4.68/ 5.51 | 5.77/6.47 |
| Study Day 8  (low sulfate day) | 7.69 | 2.75 | 1.23 | 2.40/1.31 | 6.08/6.54 |

* Air quality data obtained from regional monitor

** Predicted concentration of sulfate anions in breath condensate based on exposure and calculated value (see “Breath Sulfate Analysis” in additional file for model assumptions) versus measured concentration by ion chromatography.
